# Effect of omega-3 fatty acid diet on prostate cancer progression and cholesterol efflux in tumor-associated macrophages—dependence on GPR120

**DOI:** 10.1038/s41391-023-00745-4

**Published:** 2023-10-23

**Authors:** Pei Liang, Susanne M. Henning, Tristan Grogan, David Elashoff, Jonathan Said, Pinchas Cohen, William J. Aronson

**Affiliations:** 1 Department of Urology, David Geffen School of Medicine, University of California Los Angeles, Los Angeles, CA, USA.; 2 Department of Medicine Statistics Core, David Geffen School of Medicine, University of California Los Angeles, Los Angeles, CA, USA.; 3 Department of Pathology, David Geffen School of Medicine, University of California Los Angeles, Los Angeles, CA, USA.; 4 Leonard Davis School of Gerontology, University of Southern California, Los Angeles, CA, USA.; 5 VA Medical Center Greater Los Angeles Healthcare System, Los Angeles, CA, USA.

## Abstract

**BACKGROUND:**

Preclinical and clinical translational research supports the role of an ω-3 fatty acid diet for prostate cancer prevention and treatment. The anti-prostate cancer effects of an ω-3 diet require a functional host g-protein coupled receptor 120 (GPR120) but the underlying effects on the tumor microenvironment and host immune system are yet to be elucidated.

**METHODS:**

Friend leukemia virus B (FVB) mice received bone marrow from green fluorescent protein (GFP) labeled GPR120 wild-type (WT) or knockout (KO) mice followed by implanting Myc-driven mouse prostate cancer (MycCap) allografts and feeding an ω-3 or ω-6 diet. Tumor associated immune cells were characterized by flow cytometry, and CD206+ tumor infiltrating M2-like macrophages were isolated for gene expression studies. MycCap prostate cancer cell conditioned medium (CM) was used to stimulate murine macrophage cells (RAW264.7) and bone marrow-derived (BMD) macrophages to study the effects of docosahexanoic acid (DHA, fish-derived ω-3 fatty acid) on M2 macrophage function and cholesterol metabolism.

**RESULTS:**

The bone marrow transplantation study showed that an ω-3 as compared to an ω-6 diet inhibited MycCaP allograft tumor growth only in mice receiving GPR120 WT but not GPR120 KO bone marrow. In the ω-3 group, GPR120 WT BMD M2-like macrophages infiltrating the tumor were significantly reduced in number and gene expression of cholesterol transporters Abca1, Abca6, and Abcg1. RAW264.7 murine macrophages and BMDMs exposed to MycCaP cell CM had increased gene expression of cholesterol transporters, depleted cholesterol levels, and were converted to the M2 phenotype. These effects were inhibited by DHA through the GPR120 receptor.

**CONCLUSION:**

Host bone marrow cells with functional GPR120 are essential for the anticancer effects of dietary ω-3 fatty acids, and a key target of the ω-3 diet are the M2-like CD206+ macrophages. Our preclinical findings provide rationale for clinical trials evaluating ω-3 fatty acids as a potential therapy for prostate cancer through inhibition of GPR120 functional M2-like macrophages.

## INTRODUCTION

In numerous preclinical models, dietary ω-3 fatty acids (FAs) from fish oil delayed the development and progression of androgen-sensitive and castrate resistant prostate cancer [1–8]. Epidemiologic studies, however, report varying results on the risk of overall and aggressive prostate cancer [9–15]. In a prospective trial in men on active surveillance, higher prostate tissue eicosapentaenoic acid (EPA, ω-3) levels correlated with less upgrading on subsequent biopsies [16]. Likewise, a prospective randomized pre-prostatectomy trial reported decreased proliferation (Ki-67 index) and a decreased genetic risk score in malignant tissue in the prostatectomy specimens in men on a low-fat diet with fish oil supplements as compared to a Western diet [17, 18].

In an immunocompetent mouse model for prostate cancer, the growth inhibitory effects of an ω-3 diet were dependent on the presence of functional host g-protein coupled receptor 120 (GPR120) but not tumor cell GPR120 [6]. GPR120 is a G protein–coupled receptor involved in modulation of metabolism and endocrine and immune function and is a known receptor for long-chain polyunsaturated FAs [19, 20]. The finding that the growth inhibitory effects of an ω-3 diet are dependent on host GPR120 but not tumor cell GPR120 suggests there may be a dietary effect on the host immune system [6]. In the present study, we investigated this hypothesis using a bone marrow transplant model. We implanted MycCaP allograft tumors into immune cell-depleted (radiated) mice, infused green fluorescent protein (GFP) labeled bone marrow from either GPR120 wild-type or knockout mice, and fed the mice an ω-3 or ω-6 diet. Herein we report that the anticancer effects of the ω-3 diet are dependent on GPR120 functional bone marrow and there was a decreased number of bone marrow-derived M2-like macrophages in the tumor microenvironment.

M2-like macrophages play a critically important role in prostate cancer progression and metastases [21, 22]. At the present time there are no therapies that target M2-like macrophages, though this area is under intense investigation [23, 24]. Cancer cells are known to convert macrophages to the M2 phenotype to support cancer progression [25]. Emerging data from studies in ovarian and lung cancer suggest a key underlying mechanism promoting the M2 phenotype is cancer cell-induced depletion of macrophage cholesterol levels [26, 27]. We therefore investigated whether prostate cancer cells exhibit similar effects by increasing cholesterol efflux in tumor associated macrophages (TAMs) and if this is a potential target for ω-3 FAs from fish oil.

## MATERIALS AND METHODS

### Chemicals and reagents

Docosahexaenoic acid (DHA, ω-3 FAs) was obtained from Cayman Chemical (Ann Harbor, MI, USA). RPMI and DMEM media and fetal bovine serum were purchased from Invitrogen (Carlsbad, CA, USA); mouse IL-4 from Sigma Chemical (St Louis, MO, USA).

### Cell lines

The murine macrophage cell line RAW264.7 was purchased from American Type Culture Collection (Manassas, VA, USA). Mouse prostate cancer cell line MycCap was a gift from Dr. Lily Wu (UCLA, CA). All cell lines were used within 15 passages after thawing. Mycoplasma in the cell lines are being tested by PCR according to the manufacture’s protocol (Applied Biological Materials Inc., Richmond, BC, Canada).

### Development of EGFP transgenic FVB and EGFP x GPR120^−/−^ (GPR120 KO) mice

The transgenic enhanced green fluorescent protein (EGFP) FVB mice (FVB Cg-Tg (CAG-EGFP) B5Nagy/J) were purchased from Jackson Laboratory (Bar Harbor, ME). FVB Cg-Tg (CAG-EGFP) B5Nagy/J have expression of EGFP directed to widespread tissues by the CMV-IE enhancer/chicken β-actin/rabbit β-globin hybrid promoter and are useful as a source of fluorescently marked cells. Male and female heterozygous EGFP mice were crossed to derive homozygous EGFP mice. To generate EGFP labeled GPR120^−/−^ mice, female homozygous EGFP mice were crossed with male GPR120^−/−^ mice to generate homozygous EGFP/homozygous GPR120^−/−^ mice. GPR120^−/−^ mice were generated by the Jackson Laboratory as described previously [6].

### Genomic DNA extraction and genotyping PCR amplification

Genomic DNA was extracted and amplified as previously described [6] (primer list in Supplementary Tables 1, 2).

### Animal husbandry, feeding protocol, bone marrow transfer, and MycCaP allograft tumors

Male FVB mice (5 weeks old) were obtained from Jackson Laboratory (Bar Harbor, ME, USA). The experiments were approved by the UCLA Animal Research Committee, and animals were cared for in accordance with institutional guidelines. The following mice [FVB wild-type (WT-R = wild-type recipient) (*n*= 12 or 15 for ω-6 diet; *n*= 13 or 16 for ω-3 diet) or GPR120 knockout (KO-R = GPR120 knockout recipient) mice (*n*= 5 or 7 for ω-6 diet; n= 5 or 7 for ω-3 diet)] were subjected to X-ray irradiation (9 Gray for 6 min and 40 s) at 6 weeks of age. The following day, tail vein injection with 200 μl of 1 × 10^7^ GFP labeled bone marrow cells (1.5 × 10^6^ cells/mouse) isolated from EGFP wild-type or EGFP × GPR120^−/−^ mice respectively. Three weeks after tail vein injection, mice were acclimated on a standard AIN- 93G diet (DYETS, Bethlehem, PA) for 7-days, followed by subcutaneous injection of 5 × 10^5^ MycCaP cells (prostate cancer cell line from Hi-Myc transgenic mice) into the flank of wild-type (WT) and GPR120 KO mice as previously described [6]. The diets were prepared by DYETS, Inc. (Bethlehem, PA) as previously described [8]. Both diets provided 30% of energy (kcal) from fat with the fat source being either fish oil (ω-3 diet) or corn oil (ω-6 diet). When Myc-Cap tumors reached a size of 30–50 mm^3^, mice were assigned to either ω-3 or ω-6 diet based on matching tumor size. Mice were sacrificed 6 weeks after the MycCaP cells were injected. Tumor tissue was weighed and rinsed with cold phosphate buffered saline (PBS). 100 mg of the tumor tissue was snap-frozen in liquid nitrogen, and the remaining tissue used for flow cytometry and immune cell isolation. The mouse study including four subgroups, described above, was performed once.

### Flow cytometry analysis of immune cells

To quantify immune cells, single-cell suspensions were prepared from tumor tissue and were incubated with the following antibodies in 1:100 dilution: CD45-PE (hematopoietic cells, eBioscience, San Diego, CA), F4/80-PE-Cy7 (macrophages, eBioscience, San Diego, CA), CD11b-FITC (monocyte/macrophages, BD Biosciences San Jose, CA), CD206-APC (M2 type macrophages, Biolegend, San Diego, CA),CD68-PerCP-Cy5.5 for macrophages (M1 type macrophages,Biolegend, San Diego, CA), CD45-PE, F4/80-PE-Cy7, CD11b-FITC, Gr-1-APC (myeloid cells, BD Biosciences San Jose, CA) for myeloid derived suppressor cells (MDSCs) and Neutrophils, CD45-PE, CD4-FITC (CD4 T cells, BD Biosciences San Jose, CA), CD8-APC (CD8 T cells, BD Biosciences San Jose, CA), B220 (B cells, Biolegend, San Diego, CA) for T and B cells. Cells were washed with PBS before analysis on the BD LSR-II flow cytometer (Beckman Coulter), GFP+ cells were gated for analysis of immune cells as previously described [6].

### Magnetic cell sorting of CD206+ macrophages cells

CD206+ macrophages were isolated from single-cell suspensions from tumor tissue using CD206-APC primary antibody and anti-APC magnetic beads with miniMACS (MiltenyiBiotec, Auburn, CA, USA) according to manufacturer’s protocol [6]. The CD206+ macrophage fraction contained >95% macrophages, as confirmed by flow cytometry.

### mRNA isolation and reverse transcription-PCR (RT-PCR), semiquantitative and quantitative real-time PCR

Total RNA was isolated and RT-PCR performed as previously described [6].

### Generation of MycCap conditioned medium (CM)

1 × 10^7^ MycCap cells were seeded into 150 mm dish and 25 ml of medium containing 4% FBS was added, and the supernatant was collected 72 h later. The supernatant was filtered with 22 uM filter, collected as CM and storage in −80 °C.

### Generation of L929 conditioned media and bone marrow-derived macrophages (BMDMs)

L929 conditioned media (CM) was generated and BMDMs derived as previously described [28].

### Cholesterol measurement

Total cell cholesterol content was measured in Raw264.7 and BMDMs using the Amplex Red Cholesterol Assay kit (ThermoFisher Scientific), according to the manufacturer’s instructions. Briefly, 10^6^ cells were harvested and washed 3 times with cold PBS prior to lysis. 200 μL of chloroform: isopropanol: NP-40 (7:11:0.1) mixture was then added and centrifuged 10 min at 15,000 × *g*. The liquid (organic phase) was transferred to a new tube and air dry at 50 °C to remove the chloroform. Samples were put under vacuum for 30 min to remove the trace amounts of organic solvent and the dried lipids were dissolved in 200 μL of 1X Assay Diluent with vortexing until the solution is homogenous. 1–50 μL of extracted sample were diluted and used for assay.

### Statistical analysis

**Fig. 1:**
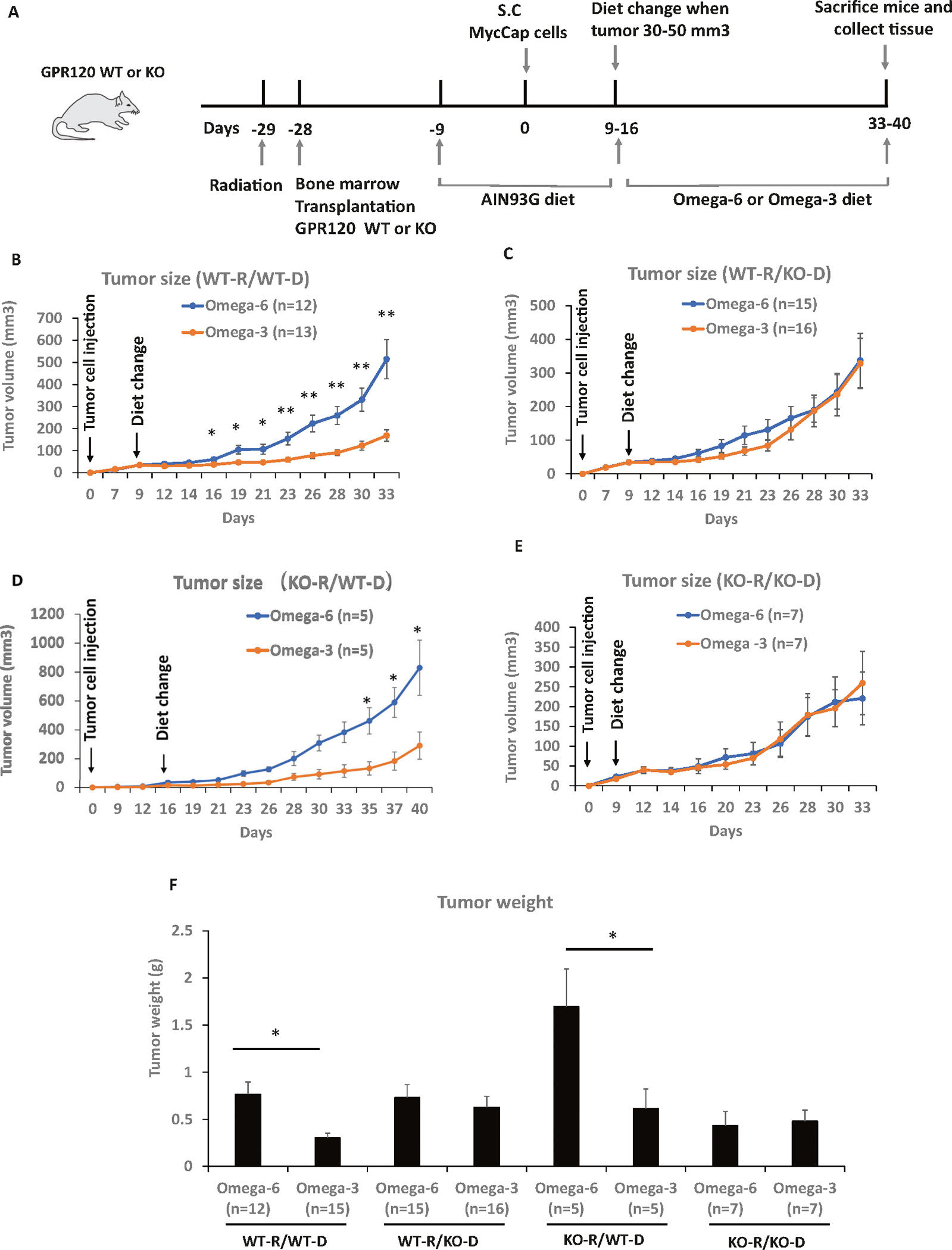
An ω-3 diet as compared to an ω-6 diet inhibited tumor growth in mice that received GPR120 WT bone marrow. **A** Mouse model scheme. **B–E** Tumor volume by time growth curves of MycCaP allografts grown subcutaneously in FVB wild-type (WT-R = wild-type recipient) (*n*= 12 or 15 for ω-6 diet; *n*= 13 or 16 for ω-3 diet) or GPR120 knockout (KO-R = GPR120 knockout recipient) mice (*n*= 5 or 7 for ω-6 diet; n= 5 or 7 for ω-3 diet). The donor bone marrow is either from wild-type (WT-D = wild-type donor) or GPR120 knockout (KO-D = GPR120 knockout donor) FVB mice. **F** Tumor weights at time of sacrifice. Significance was determined by Student t test. (* <0.05 and ** <0.01). Tumor growth curves were steeper in the ω-6 group compared to ω-3 group (*p* < 0.001) in (**B**) and (**D**) but not significantly different in (**C**) (*p*= 0.570) or (**E**) (*p*= 0.916).

Mouse group size was determined based on previous studies using the MycCap allograft tumor model [5, 6, 8, 28]. Quantitative measures (caloric intake, mean body weights, final tumor volume and weight, M1, M2-like macrophages, mRNA levels, etc.) were summarized by group as mean ± standard error of the mean (SEM), unless otherwise noted and formally compared between groups using the Student’s *t* test using the GraphPad Prism6.0 software (GraphPad Software, La Jolla CA). Investigators were not blinded to the diet composition. Tumor growth curves for Fig. 1 were compared between groups (ω-3/ω-6) using linear mixed effects models with terms for time, group, and the group*time interaction with a random mouse effect (to properly account for the repeated measures design of the study). In vitro experiments were performed in triplicate. *P* values < 0.05 was considered statistically significant.

## RESULTS

### The anti-cancer effect of an ω-3 diet is dependent on bone marrow cells with functional GPR120

The anti-cancer effect of an ω-3 diet was previously found to be dependent on functional GPR120 in the host [6], but the host mechanism is yet to be defined. We hypothesized that ω-3 FAs in fish oil act through GPR120 functional immune cells from the bone marrow that migrate to the tumor microenvironment. To test this hypothesis we performed a bone marrow transplant experiment with FVB GPR120 WT or GPR120 KO mice and tail vein injection of GFP labeled bone marrow from GPR120 WT or GPR120 KO mice. After 3 weeks we implanted MycCap cells subcutaneously. Once the MycCap tumors reached 30–50 mm^3^, the mice were fed an isocaloric ω-3 or ω-6 diet (Fig. 1A). When irradiated GPR120 WT mice were injected with GPR120 WT bone marrow, the ω-3 diet compared with the ω-6 diet significantly inhibited tumor growth and final tumor volume (514.8 ± 88.6 mm3 in ω-6 diet versus 168.4 ± 26.5 mm3 in ω-3 diet, *p*= 0.0007) and decreased final tumor weight (0.77 ± 0.13 g in ω-6 diet versus 0.31 ± 0.05 g in ω-3 diet, *p*= 0.002) (Fig. 1B, F). However, when GPR120 KO bone marrow cells were injected into irradiated GPR120 WT mice, feeding the ω-3 diet compared to the ω-6 diet did not reduce tumor growth and final tumor volume (337.2 ± 80.9 mm3 in ω-6 diet versus 328.5 ± 74.8 mm3 in ω-3 diet) and final tumor weight (0.73 ± 0.14 g in ω-6 diet versus 0.63 ± 0.12 g in ω-3 diet) (Fig. 1C, F). Previously, an ω-3 diet was found not to inhibit tumor growth in MycCaP tumors grown in global GPR120 KO mice [6]. However, in the present experiment, after injecting GPR120 WT bone marrow into irradiated GRP120 KO mice, the ω-3 compared to the ω-6 diet inhibited allograft tumor growth and tumor volume (829.3 ± 190.6 mm3 in ω-6 diet versus 291.3 ± 94.1 mm3 in ω-3 diet, *p*= 0.035) and tumor weight (1.70 ± 0.40 g in ω-6 diet versus 0.61 ± 0.21 g in ω-3 diet, *p*= 0.04), (Fig. 1D, F). When GPR120 KO bone marrow cells were injected into GPR120 KO recipient mice, feeding the ω-3 diet compared to the ω-6 diet did not reduce tumor growth and final tumor volume (220.6 ± 66.7 mm^3^ in ω-6 diet versus 259.3 ± 79.7 mm^3^ in ω-3 diet) and tumor weight (0.43 ± 0.15 g in ω-6 diet versus 0.48 ± 0.12 g in ω-3 diet) (Fig. 1E, F). Throughout the experiments there was no difference in mouse body weight or food intake between the groups (Supplementary Fig. 1).

### An ω-3 diet reduced the number of GPR120 wild-type bone marrow-derived M2 macrophages in MycCaP allografts

**Fig. 2:**
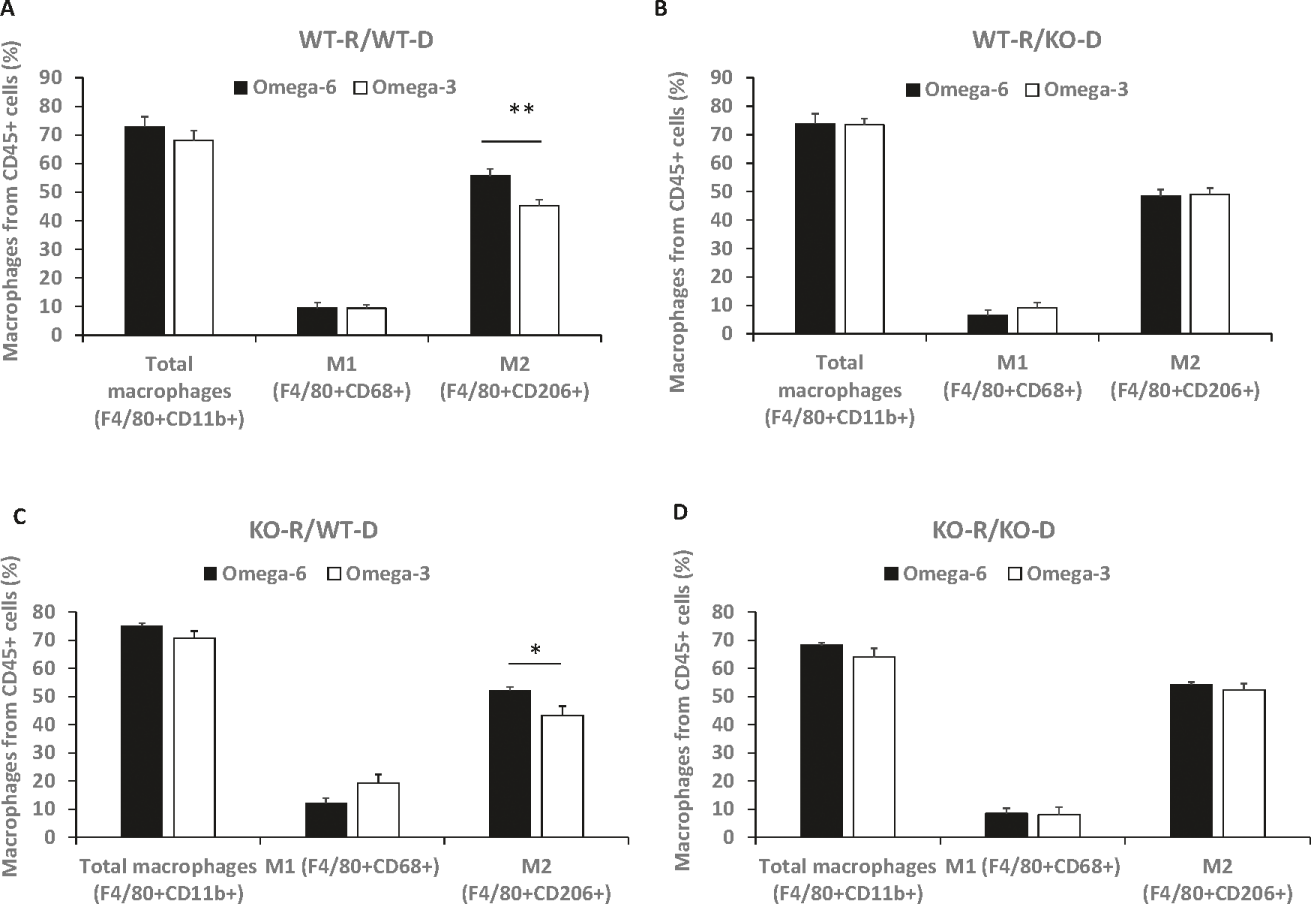
The ω-3 as compared to the ω-6 diet decreased M2 macrophages in tumor tissue in mice that received GPR120 WT bone marrow. **A–D** Percentage of macrophage subtypes from CD45+ cells in tumor tissue as determined by flow cytometry. Cells displayed in Fig. 2 are GFP+. Total macrophages = F4/80 + CD11b+, M1 macrophages = F4/80 + CD68+, and M2 macrophages = F4/80 + CD206 +. WT-R = wild-type recipient, KO-R = GPR120 knockout recipient, WT-D = wild-type donor, KO-D = GPR120 knockout donor. WT-R: *n*= 12 or 15 for ω-6 diet; *n*= 13 or 16 for ω-3 diet; KO-R: *n*= 5 or 7 for ω-6 diet; *n*= 5 or 7 for ω-3 diet. Significance determined by Student’s *t* test (* <0.05 and ** <0.01).

**Fig. 3:**
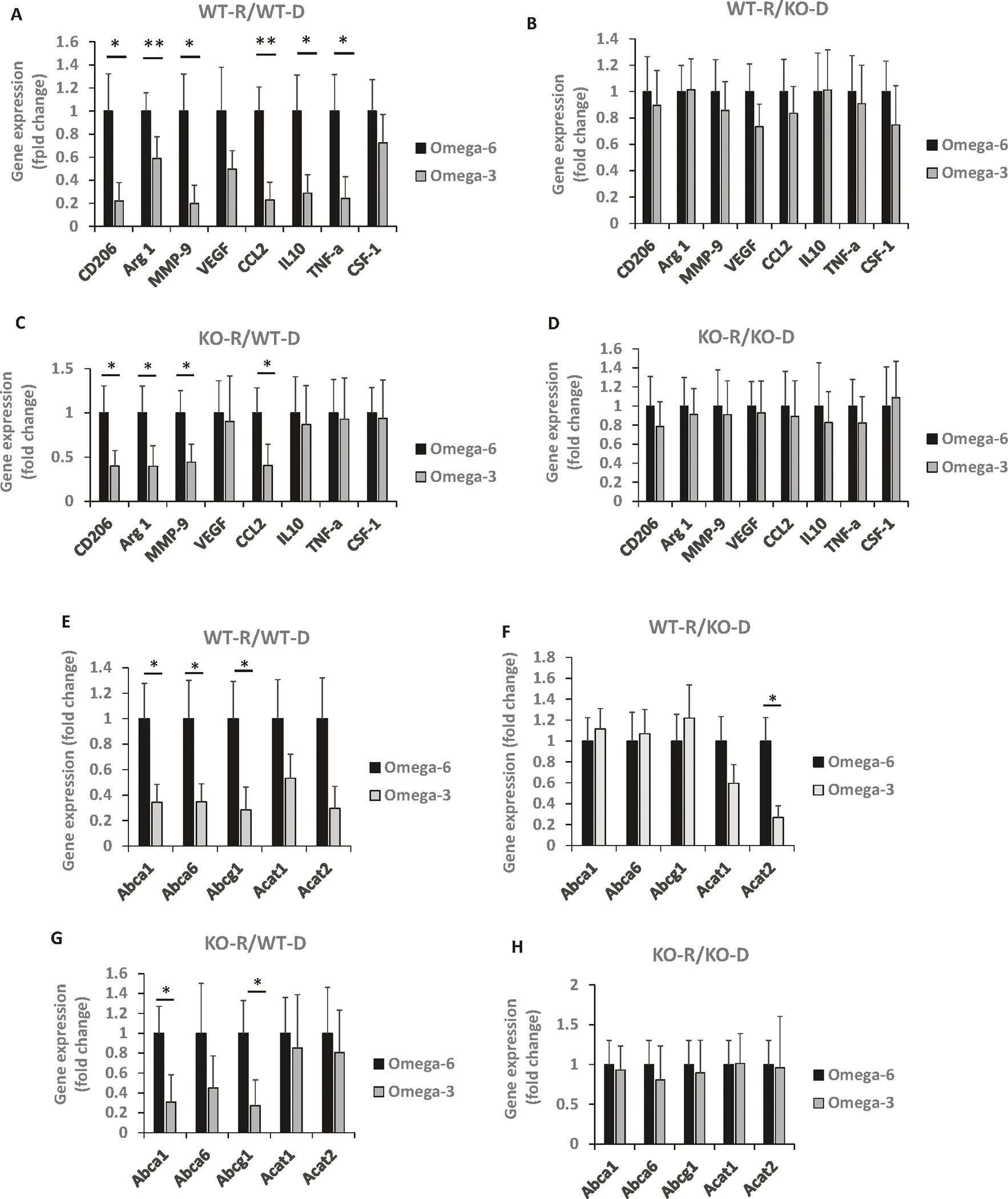
The ω-3 diet regulated cholesterol efflux and polarization and function of macrophages in tumor tissue in mice that received GPR120 WT bone marrow. For Figures (**A**–**H**), macrophages were isolated from the MycCaP tumors as described in the methods section. **A**–**D** Gene expression of M2 markers in macrophages isolated from MycCaP allografts. **E**–**H** Gene expression of cholesterol metabolism enzymes in macrophages isolated from MycCaP allografts. WT-R = wild-type recipient, KO-R = GPR120 knockout recipient, WT-D = wild-type donor, KO-D = GPR120 knockout donor. WT-R: *n*= 12 or 15 for ω-6 diet; *n*= 13 or 16 for ω-3 diet; KO-R: *n*= 5 or 7 for ω-6 diet; *n*= 5 or 7 for ω-3 diet. Significance determined by Student’s *t* test (* <0.05 and ** <0.01).

Previously, we reported that feeding an ω-3 diet decreased M2-like macrophage tumor infiltration [6]. For the bone marrow transplantation experiments described above, we sought to determine the effect of the ω-3 vs. ω-6 diet on bone marrow-derived immune cells infiltrating the MycCap allograft tumors and the dependence on GPR120. By flow cytometry, 70–80% of the hematopoietic, CD45+ cells in the MycCaP tumors were green fluorescent protein positive (GPF+) cells and thus derived from the infused bone marrow (data not shown). Flow cytometry was performed on the tumor GFP+/CD45+ cells to determine the populations of bone marrow-derived immune cells in the tumor tissue. In the recipient GPR120 WT mice that received GPR120 WT bone marrow, the number of F4/80 + CD206 + M2 polarized macrophages was significantly decreased from 55.8 ± 2.3% (ω-6 diet) to 45.2 ± 2.1% (ω-3 diet) (*p*= 0.0036) (Fig. 2A). However, there was no significant difference between the ω-3 and ω-6 groups in the number of F4/80 + CD206 + M2 polarized macrophages in the tumors of the recipient GPR120 WT mice that received GPR120 KO bone marrow (Fig. 2B). In the GPR120 KO mice that received GPR120 WT bone marrow, the number of F4/80 + CD206 + M2 polarized macrophages was significantly decreased from 52.2 ± 1.1% (ω-6 diet) to 43.2 ± 3.3% (ω-3 diet) (*p*= 0.035) (Fig. 2C). However, there was no significant difference between the ω-3 and ω-6 groups in the number of F4/80 + CD206 + M2 polarized macrophages in the tumors in the GPR120 KO mice that received GPR120 KO bone marrow (Fig. 2D). There was no significant difference between the ω-3 or ω-6 diet groups in the other immune cells infiltrating the allografts such as F4/80 + CD11b+ total macrophages, F4/80 + CD68 + M1 macrophages (Fig. 3A–D), CD11b + Gr1+ MDSCs, F4/80-CD11b + Gr1+ neutrophils, CD8^+^ or CD4 + T cells and B220 + B cells (Supplementary Fig. 2).

We isolated CD206+ (M2) macrophages from the MycCaP tumors using magnetic beads to study the effect of the ω-3 diet on gene expression of M2-like macrophage markers, Gene expression of CD206, Arg1, MMP-9, CCL2, IL-10 and TNF-α were significantly decreased in isolated CD206+ cells from allograft tissue in the ω-3 group compared to the ω-6 group from recipient GPR120 WT or KO mice that received GPR120 WT bone marrow (Fig. 3A, C), but not from recipient GPR120 WT or KO mice that received GPR120 KO bone marrow (Fig. 3B, D). Thus, the ω-3 diet only reduced the CD206+ (M2) macrophages infiltrating the MycCaP allografts when the donor bone marrow was GPR120 wild-type.

### An ω-3 diet decreased expression of cholesterol transporter genes in GPR120 wild-type M2 macrophages isolated from MycCaP allografts

Based on recent studies reporting that cholesterol efflux from TAMs leads to cancer progression, we sought to determine if the ω-3 diet inhibits cholesterol efflux from tumor infiltrating M2-like macrophages [26, 27, 29]. We found that the ω-3 diet as compared to the ω-6 diet significantly reduced gene expression of cholesterol transporters Abca1, Abca6 and Abcg1 in M2 (CD206+) macrophages isolated from MycCaP allograft tumors in irradiated GPR120 WT and KO mice given GPR120 WT bone marrow (Fig. 3E, G). However, there was no significant effect on cholesterol transporters in M2 (CD206+) macrophages isolated from MycCaP tumors in GPR120 WT or KO mice given GPR120 KO bone marrow (Fig. 3F, H).

### Docosahexaenoic acid (DHA) decreased MycCaP conditioned media-induced cholesterol efflux from M2 macrophages

**Fig. 4:**
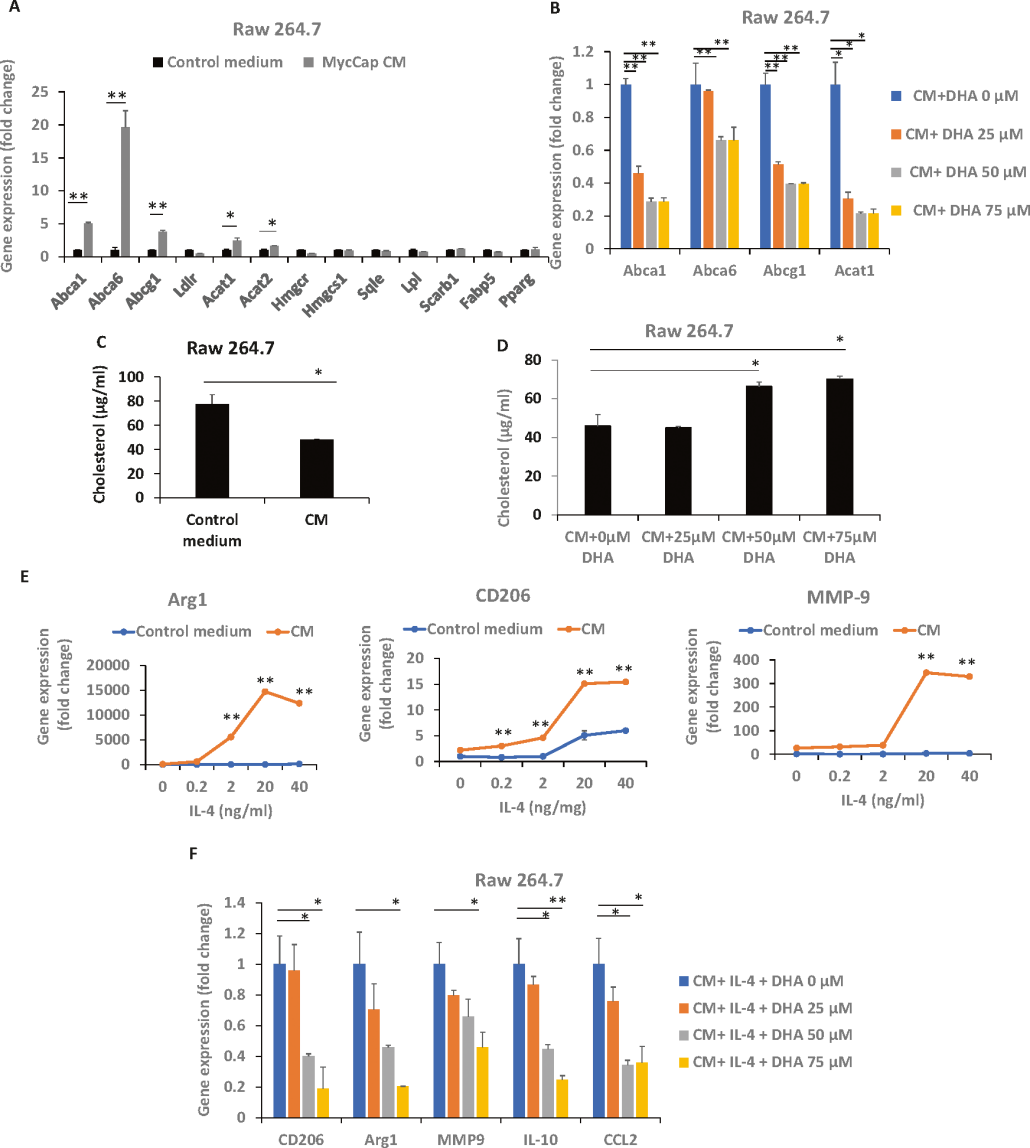
MycCaP conditioned medium induced cholesterol efflux and conditioned medium induced gene expression of M2 markers was inhibited by DHA (ω-3 fatty acid). **A, C** MycCaP conditioned medium effects on cholesterol transport and cholesterol levels in RAW 264.7 macrophages. **B**, **D** DHA inhibitory effects on cholesterol metabolism enzymes and cholesterol levels in MycCaP conditioned medium treated RAW 264.7 macrophages. **E** MycCaP conditioned medium induced expression of M2 markers in RAW 264.7 cells in response to IL-4 treatment. **F** DHA effects on MycCaP conditioned medium induced expression of M2 markers in response to IL-4 treatment (*n*= 3). Significance determined by Significance determine by Student’s *t* test (* <0.05 and ** <0.01).

Recent studies in ovarian and lung cancer reported that cancer cells are able to induce transition of TAMs to the M-2 phenotype by altering macrophage lipid metabolism [26, 27]. In the study by Goossens P. et al., co-culture of the ovarian cancer cell line ID-8 with bone marrow-derived macrophages (BMDMs) increased cholesterol efflux in lipid rafts of BMDMs and increased IL-4-mediated BMDM reprogramming [26]. To examine the role of ω-3 FAs affecting prostate cancer-induced alterations in macrophage cholesterol metabolism we utilized an in-vitro model in which Raw 264.7 macrophages were cultured with MycCaP cell CM. MycCaP CM significantly upregulated gene expression of cholesterol transporters Abca1, Abca6 and Abcg1 and cholesterol synthesis-related genes Acat1 and Acat2 and reduced membrane cholesterol levels in Raw 264.7 macrophages, and these effects (excluding Acat2 expression) were inhibited by the ω-3 fatty acid DHA (Fig. 4A–D). MycCaP CM also induced Raw 264.7 macrophages to have characteristics of M2 macrophages (increased expression of Arg 1, CD206, and MMP9) (Fig. 4E). DHA inhibited expression of M2 markers (CD206, Arg 1, MMP9, IL10, and CCL2) in Raw 264.7 macrophages exposed to MycCaP CM and IL-4 (Fig. 4F).

**Fig. 5:**
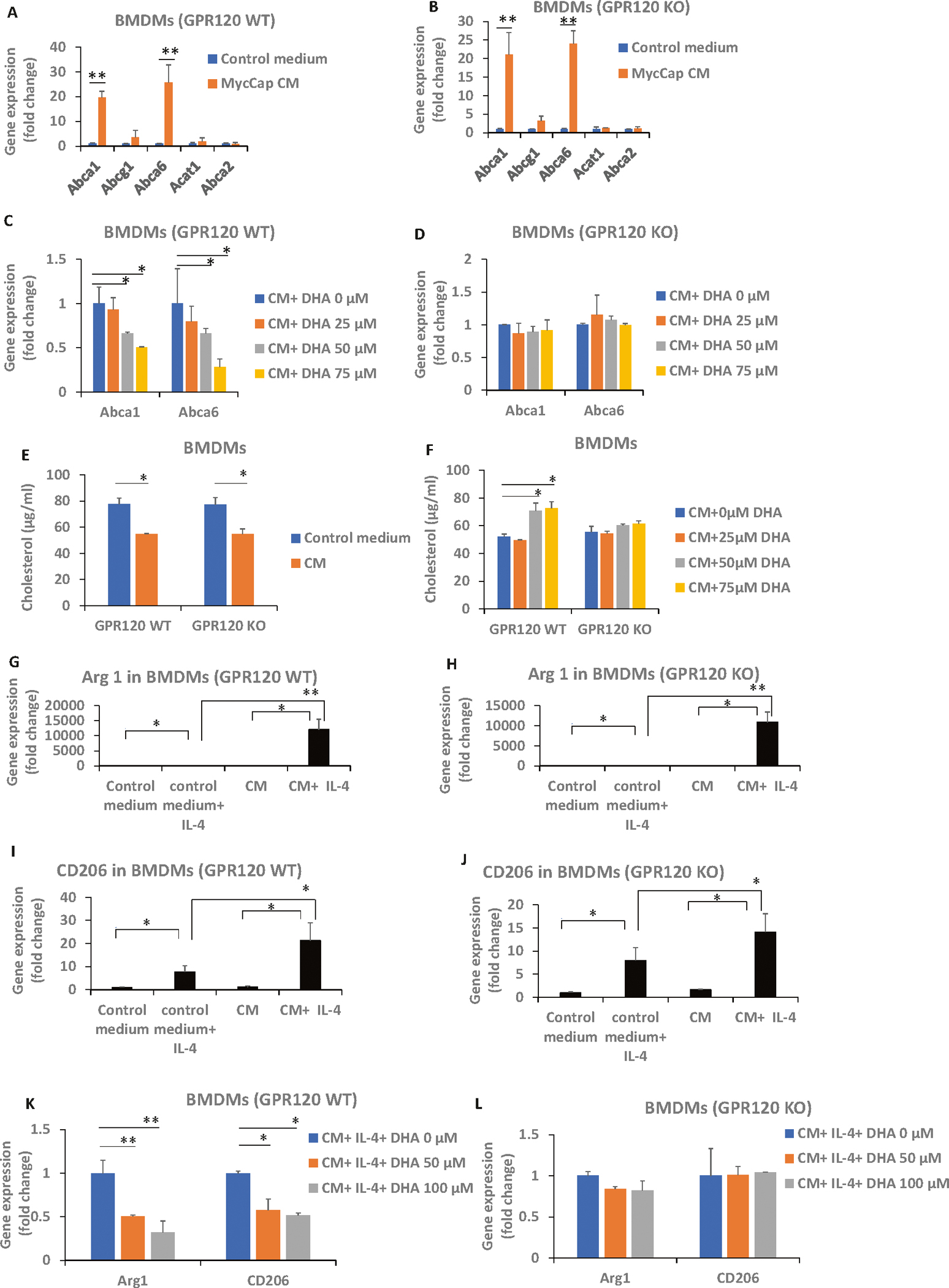
MycCaP conditioned medium induced cholesterol efflux and conditioned medium induced gene expression of M2 markers was inhibited by DHA in bone marrow derived macrophages (BMDMs) with a functional GPR120 receptor. Bone marrow derived macrophages were generated as described in the methods section. **A**, **B** MycCaP conditioned media induced expression of cholesterol transporters in GPR120 WT and KO BMDMs. **C**, **D** DHA inhibition of cholesterol efflux enzymes induced by MycCaP conditioned media in GPR120 WT and KO BMDMs. **E** Effect of MycCaP conditioned media on cholesterol levels in GPR120 WT and KO BMDMs. **F** Effect of DHA on cholesterol levels in MycCaP conditioned media treated GRP120 WT and KO BMDMs. **G**–**J** Effect of MycCaP conditioned media on M2 markers in IL-4 treated GPR120 WT and KO BMDMs. **K**, **L** Effect of DHA on expression of M2 markers in MycCaP conditioned media and IL-4 treated GPR120 WT and KO BMDM’s. Significance determined by Student’s *t* test (* <0.05 and ** <0.01).

To determine if the inhibitory effects of DHA on cholesterol efflux from M2-like macrophages is mediated through GPR120, we utilized an ex-vivo assay using BMDMs from GPR120 WT and KO mice. MycCap CM increased expression of the cholesterol transporters Abca1 and Abca6 and lowered membrane cholesterol levels in GPR120 WT and KO BMDMs (Fig. 5A, B, E). Addition of DHA inhibited these effects in the GPR120 WT BMDMs but had no significant effect in the GPR120KO BMDMs (Fig. 5C, D, F). MycCap CM increased IL-4 induced Arg 1 and CD206 expression in GPR120 WT and KO BMDMs and this effect was inhibited by DHA in the GPR120 WT BMDMs but not the GPR120 KO BMDMs (Fig. 5G–L). Thus, the inhibitory effects of DHA on cholesterol efflux and reduction in cholesterol levels in M2-like macrophages are dependent on a functional GPR120 receptor.

## DISCUSSION

The anti-prostate cancer effects of dietary ω-3 FAs were previously found to be dependent on functional GPR120 in the host, however, the underlying host mechanisms were not defined. We hypothesized that dietary ω-3 FAs inhibit prostate cancer progression through effects on immune cells derived from bone marrow with functional GPR120 that migrate to the tumor microenvironment. To test this hypothesis, we grew MycCaP allografts in a bone marrow transplant model using GFP-labeled bone marrow from either GPR120 wild-type or knockout mice and fed the mice ω-3 or ω-6 FA diets. We found that the ω-3 diet only inhibited MycCap allograft growth when the recipient mice were injected with bone marrow from GPR120 WT donor mice. Among the GFP-labeled immune cells infiltrating the MycCaP allografts, only the M2-like macrophages (CD206 + ) were reduced in number in the ω-3 vs the ω-6 diet groups. Likewise, gene expression of M2 markers in the GFP+ tumor infiltrating CD206+ macrophages, known to be associated with prostate cancer progression, were only reduced when the M2-like macrophages had functional GPR120. In summary, host bone marrow cells with functional GPR120 are essential for the anticancer effects of dietary ω-3 FAs, and a key target of the ω-3 diet is the M2-like CD206+ macrophages.

The mechanism whereby prostate cancer cells convert macrophages to the M2 phenotype to support progression and metastasis is yet to be elucidated. ABCA1, ABCA6 and ABCG1 are cholesterol transporters and suppress the accumulation of cholesterol in macrophages [30, 31]. Emerging data suggests that cancer cells secrete factors that activate cholesterol transporters in macrophages such as ABCA1 ABCA6 and ABCG1 to increase cholesterol efflux [26, 27]. Depletion of cholesterol from TAMs increases sensitivity to IL4 and promotes polarization to M2-like macrophages to support cancer progression [26]. Based on these emerging data, we sought to determine if dietary ω-3 FAs inhibit prostate cancer induced cholesterol efflux from macrophages and conversion to the M2 phenotype, and if these effects are dependent on GPR120. We found that MycCaP CM significantly upregulated gene expression of cholesterol transporters and reduced cholesterol levels in Raw 264.7 macrophages and bone marrow-derived M2 macrophages. The MycCaP CM also increased M2 phenotypic characteristics in response to IL-4. These MycCaP CM effects were inhibited by DHA treatment, but only in the presence of a functional GPR120. Thus, in pre-clinical models, it appears that dietary ω-3 FAs inhibit prostate cancer-induced TAM cholesterol efflux and M2 macrophage polarization and function through GPR120. Of interest, El-Kenawi et al. reported that macrophage depletion of cholesterol reduced androgen levels within prostate tumors and restricted AR nuclear localization [29]. Thus, dietary ω-3 FAs may have the potential to reduce intratumoral androgen levels in prostate cancer through inhibition of M2 macrophage cholesterol efflux.

Yet to be determined is if the anticarcinogenic effects of an ω-3 diet in pre-clinical and early phase clinical studies will translate to efficacy in patients, either for prevention or treatment of prostate cancer. A search on Clinicaltrials.gov of omega-3, fish oil, and prostate cancer references four ongoing trials in men with prostate cancer in varying stages of their disease. The group at CHU de Quebec-Université Laval is evaluating a fish oil supplement enriched with a marine-derived ω-3 FA (EPA) for men undergoing radical prostatectomy (NCT02333435) and men with biochemical recurrence (NCT03753334). The group at Cedar Sinai Medical Center is evaluating walnuts (high in plant-based ω-3 FAs) in the pre-prostatectomy setting (NCT03824652). Our group is evaluating a low ω-6 diet combined with a fish oil supplement for men on active surveillance (NCT02176902). Hopefully, these early phase trials will yield positive findings that will ultimately benefit our patients. Based on a prior pre-prostatectomy trial suggesting increased GPR120 expression in the prostate positively correlates with the antiproliferative effects of dietary ω-3 FAs, possibly a patient’s GPR120 status will be predictive of response to dietary ω-3 FAs, thus allowing a precision approach to dietary therapy [6]. It is of great interest that the anticancer effects of ω-3 FAs are dependent on the tumor microenvironment as opposed to direct effects on the tumor. Thus, combining a dietary therapy that targets GPR120 in the microenvironment with tumor-targeting therapies may also be beneficial.

## Supplementary Material

Supplementary Information

## Data Availability

The data sets generated during the present study are available from the corresponding author upon reasonable request.
