# Supplementary material for: Effect of omega-3 fatty acid diet on prostate cancer progression and cholesterol efflux in tumor-associated macrophages—dependence on GPR120
Source: Prostate Cancer Prostatic Dis. Author manuscript; Available in PMC 2025 Jun 1. (PMC11035487; doi:10.1038/s41391-023-00745-4)
Supplement: Supplementary Information [file NIHMS1947766-supplement-Supplementary_Information.pdf]

Supplementary Figure 1

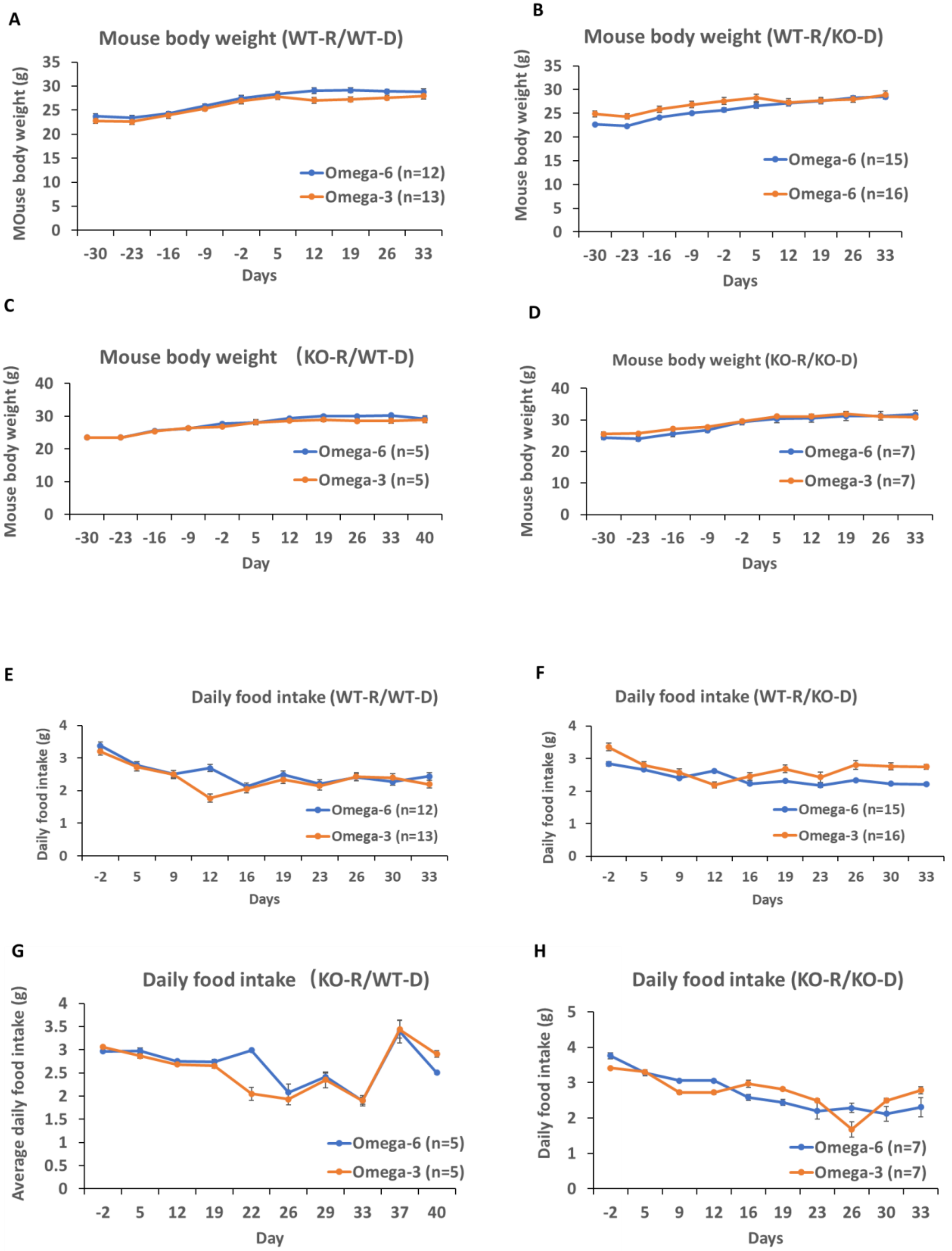

Supplementary Figure 1: Mouse body weight and food intake during the bone marrow transplant experiment. A-D) Mouse body weight. E-H) Daily food intake. WT-R = wild-type recipient, KO-R = GPR120 knockout recipient, WT-D = wild-type donor, KO-D = GPR120 knockout donor.

Supplementary Figure 2

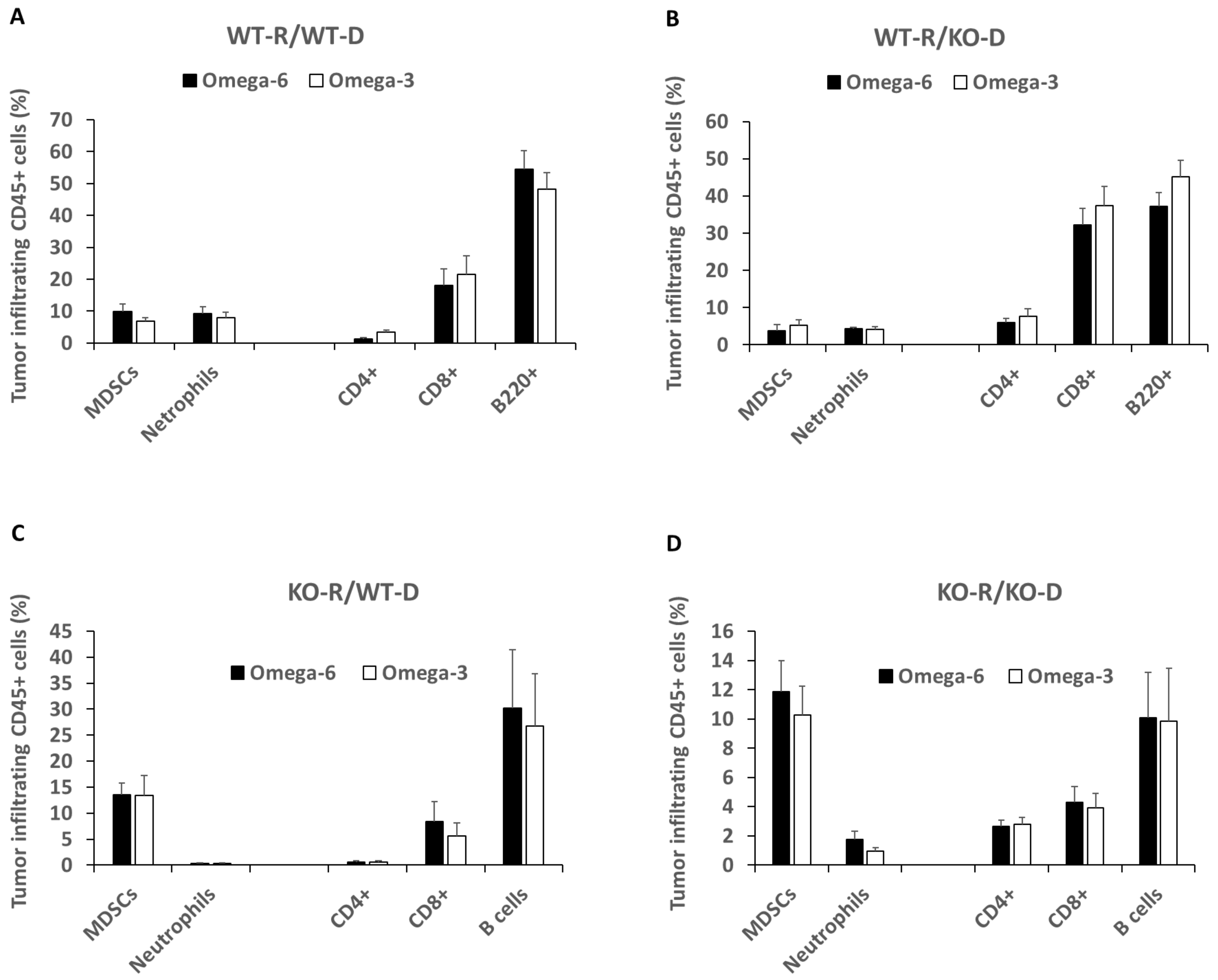

Supplementary Figure 2: GFP+ CD45+ cells infiltrating the allograft tumors. WT-R = wild-type recipient, KO-R = GPR120 knockout recipient, WT-D = wild-type donor, KO-D = GPR120 knockout donor.

Supplementary Table 1： Primer list for genotyping

**Hi-Myc**	**Gene of interest primer mix**	**Forward: 5'-CCTCCAGCAGAAGGTGATCC-3'**<br>**Reverse: 5'-AGCGACTCTGAGGAGGAACA-3'**
	**Internal control primer mix**	**Forward: 5'-AATCGGAGAAGCAGGACCTTATC-3'**<br>**Reverse: 5'-TCCCGAAGTTGACATCAAACC-3'**
	**Gene of interest probe**	**5'-GCCTGCCTCTTTTCCACAGA-3', probe label: FAM**
	**Internal control probe**	**5'-AGTGAAGTCCTAATTCC-3', probe label: VIC**
**GFP**	**Gene of interest primer mix**	**Forward: 5'-AGT GCT TCA GCC GCT ACC-3'**<br>**Reverse: 5'- GAA GAT GGT GCG CTC CTG -3'**
	**Internal control primer mix**	**Forward: 5'- CAC GTG GGC TCC AGC ATT -3'**<br>**Reverse: 5'- TCA CCA GTC ATT TCT GCC TTT G -3'**
	**Gene of interest probe**	**5'- TTC AAG TCC GCC ATG CCC GAA -3', probe label: FAM**
	**Internal control probe**	**5'- CCA ATG GTC GGG CAC TGC TCA A -3', probe label: VIC**
**GPR120**	**Gene of interest primer mix**	**Forward: CCT GAG TGT GCA CAG ACG AC**<br>**Reverse: CAT CCG AGA AGA AAG GGA AG**
	**WT Probe**	**5'-CAC ACC CTG GAC CAA GTC A-3', probe label: FAM**
	**MUT Probe**	**5'-CTC GCA CAC CAA GTC AAT CG-3', probe label: VIC**

Supplementary Table 2： Primer list for gene expression

Gene	Forward primer (5'→3')	Reverse primer (5'→3')
Mouse CD206	ATGGATTGCCCTGAACAGCA	TGTACCGCACCCTCCATCTA
Mouse Arg1	CAGCACTGAGGAAAGCTGGT	ACAGACCGTGGGTTCTTCAC
Mouse TNF-α	CACCACGCTCTTCTGTCTAC	CCATAGAACTGATGAGAGGG
Mouse CCL2	GGCTGGAGAGCTACAAGAGG	GGTCAGCACAGACCTCTCTC
Mouse CCL22	TGGACCTCAAAATCCTGCCG	CAGGTCCTCCTCCCTAGGAC
Mouse VEGF	ACTGGACCCTGGCTTTACTG	CTCTCCTTCTGTCGTGGGTG
Mouse MMP-9	GACGACATAGACGGCATCCA	TGTTGTTCAGTTGTGGTGGT
Mouse IL-10	TAGAGCTGCGGACTGCCT	TTTCCGATAAGGCTTGGC
Mouse CSF-1	TGTTCTACAAGTGGAAGTGGAGG	TGGTGAGGGGTCATAGAA
Mouse Abca1	GCAGAAACAGTAGCAGCAC	TCTCCTCCTCTGCCTCCAC
Mouse Abca6	CGGAGAGCAGCCACCTACTT	ACGTGCACACTTAGCTCCTT
Mouse Abcg1	AAGGTCTCCAATCTCGTGCC	CCCTGATGCCACTTCCATGA
Mouse Acat1	CCAATGCCAGCACACTGAAC	TCTACGGCAGCATCAGCAAA
Mouse Acat2	AGTGCTGGTGTCTTCCAGAAA	CTGATGCGTTCGCTGGGG
Mouse Ldlr	AGACCCAGAGCCATCGTAGT	CCACACCATTCAAACCCCCT
Mouse Hmgcr	AGAACAAGGGTTCACGCCCA	CAAAGGAAGTGACCCAGGCA
Mouse Sqle	GAGGCTACCGTGTTCTCCAG	TGGTTTCTGACAGTGGGTACG
Mouse LPl	AGCAAGACCTTCGTGGTGAT	GGCCCGATACAACCAGTCTA
Mouse Scarb1	ATGCCCCAGGTTCTTCACTAC	CCTTATCCTGGGAGCCCTTTT
Mouse Fabp5	CACGGCTTTGAGGAGTACAT	TCGGTTTTGACCGTGATGTT
Mouse Pparg	ATTAGATGACAGTGACTT	TGTCTTGGATGTCCTCGATG
